# Neuronal avalanche dynamics and functional connectivity elucidate information propagation *in vitro*

**DOI:** 10.3389/fncir.2022.980631

**Published:** 2022-09-15

**Authors:** Kristine Heiney, Ola Huse Ramstad, Vegard Fiskum, Axel Sandvig, Ioanna Sandvig, Stefano Nichele

**Affiliations:** ^1^Department of Computer Science, Oslo Metropolitan University, Oslo, Norway; ^2^Department of Computer Science, Norwegian University of Science and Technology, Trondheim, Norway; ^3^Department of Neuromedicine and Movement Science, Norwegian University of Science and Technology, Trondheim, Norway; ^4^Department of Neurology, St. Olav's Hospital, Trondheim, Norway; ^5^Department of Community Medicine and Rehabilitation, St. Olav's Hospital, Trondheim, Norway; ^6^Department of Clinical Neuroscience, Umeå University Hospital, Umeå, Sweden; ^7^Department of Computer Science and Communication, Østfold University College, Halden, Norway

**Keywords:** neural computation, microelectrode arrays, *in vitro* electrophysiology, subcritical dynamics, excitation–inhibition balance, network neuroscience, complexity

## Abstract

Cascading activity is commonly observed in complex dynamical systems, including networks of biological neurons, and how these cascades spread through the system is reliant on how the elements of the system are connected and organized. In this work, we studied networks of neurons as they matured over 50 days *in vitro* and evaluated both their dynamics and their functional connectivity structures by observing their electrophysiological activity using microelectrode array recordings. Correlations were obtained between features of their activity propagation and functional connectivity characteristics to elucidate the interplay between dynamics and structure. The results indicate that *in vitro* networks maintain a slightly subcritical state by striking a balance between integration and segregation. Our work demonstrates the complementarity of these two approaches—functional connectivity and avalanche dynamics—in studying information propagation in neurons *in vitro*, which can in turn inform the design and optimization of engineered computational substrates.

## 1. Introduction

Since the finding by Beggs and Plenz (2003) that cortical activity shows hallmarks of criticality, a dynamic state recognized as conferring many computational benefits to a system, there has been interest in understanding how critical or near-critical dynamics relate to computation in the brain (Beggs, 2008). Among the computational benefits of this state are a widened dynamic range (Kinouchi and Copelli, 2006) and increased sensitivity to inputs (Bertschinger and Natschläger, 2004). Expanding the question of criticality to *in vitro* systems has shown that neurons do not always organize into the critical state *in vitro* (Pasquale et al., 2008), though it may be possible to manipulate them to be closer to criticality (Heiney et al., 2019). However, there is increasing accord that the picture is more complicated than the cortex simply organizing into a singular critical state, with evidence showing the benefits of a “slightly subcritical” state (Priesemann et al., 2014) and demonstrating that networks can dynamically adjust their state depending on the nature of the task at hand (Wilting and Priesemann, 2018). This expanded view on criticality may give more insight into the behavior of *in vitro* networks.

The dynamics that can be supported on a network depend on how that network is connected. Because of this, network connectivity plays an important role in the emergent computational capabilities of a system; this is understood both from the perspective of how the brain produces cognition (Bressler and Menon, 2010) and from the more generalized perspective of reservoir computing (Dale et al., 2021), in which the physical properties of a dynamic system are exploited for computation. Thus it is natural to investigate the features of connectivity that accompany emergent dynamics considered beneficial for computation.

The field of network neuroscience has progressed our understanding of how neural systems are organized, from the level of populations of neurons up to the scale of brain regions, shedding light on the contribution of structural and functional organization to cognition and brain function (Bassett and Bullmore, 2017). In particular, such an approach can reveal how neuronal systems balance integration and segregation (Sporns, 2013)—in such a balanced system, densely intra-connected communities form (segregation) while global communication is maintained (integration). In practice, this can manifest in the form of hierarchical modules (Rubinov et al., 2011) or small-world networks (Downes et al., 2012), with the former characterized by self-similar clustered structures forming at different scales and the latter by low path lengths and high clustering. This type of balance in integration and segregation—where the activity of nodes in a network is coordinated but not entirely synchronous—also relates to the *complexity* of the activity, as will be defined in Section 2.2.5 (Tononi et al., 1994).

Previous modeling studies have revealed connectivity features that can facilitate the emergence of criticality in neural systems (see Heiney et al., 2019 for a review), such as scale-free-ness (Pellegrini et al., 2007) and small-worldness (Pajevic and Plenz, 2009), with different connection densities yielding different dynamics (Lin and Chen, 2005). In particular, Massobrio et al. (2015) showed that, although randomly connected networks *can* support critical dynamics, their resultant firing rates are biologically implausible, whereas scale-free and small-world networks are able to reproduce behavior observed experimentally *in vitro*.

With this theoretical evidence that connectivity structure plays such an important role in determining network dynamics, it would be valuable to observe the connectivity features that accompany different regimes of dynamic behavior in networks of neurons to better understand how structure and dynamics relate and influence one another. Furthermore, interplay between dynamics and connectivity can give us insight into how they relate to the emergent computation in a system, and thus studying the two aspects of a neural system in tandem can provide new insights into neural information processing. In addition to advancing our understanding of neural computation, this also opens the door to designing artificial intelligence models with dynamics and connectivity features similar to those observed empirically.

In this study, the electrophysiological activity of *in vitro* neuronal networks prepared at two different seeding densities (*N* = 3 high-density and *N* = 3 low-density networks) was observed using extracellular recording with a microelectrode array (MEA). The activity was characterized using two analytical approaches: analysis of the characteristics of neuronal avalanches and computation of graph theory metrics from extracted functional connectivity graphs. The relationship between these two sets of metrics was evaluated for the two different seeding densities as the networks matured over nearly a month *in vitro*. This study demonstrates that computational methods to study neuronal avalanche dynamics and network connectivity can complement each other and provide greater insight about the propagation of information in neuronal networks (Heiney et al., 2021). Our results lay a foundation upon which we can build a deeper understanding of how the topological features of the functional connectivity of neuronal networks *in vitro* relate to the emergence of different dynamical regimes, as well as how self-organized dynamics and structure relate to neural computation (Turnbull et al., 2018; Heiney et al., 2020).

## 2. Methods

This section first describes the experimental details of this study, then presents the computational methods used to analyze the electrophysiological data. The analytical methods applied here comprise two complementary approaches: the study of activity propagation through the network through the lens of cascades of activity termed “neuronal avalanches,” and the extraction and analysis of functional connectivity graphs. The former set of methods gives insight into the dynamics occurring in the network; as none of the networks observed here were seen to organize into the critical state, the focus of these methods is on the branching ratio and a measure of the complexity of activity, as other methods associated with neuronal avalanche analysis (e.g., power-law fitting and shape collapse) are only meaningful when the system is functioning very close to the critical state. The latter set of methods allows us to describe how the networks are organized to allow the propagation of activity and information. We focus on evaluating and comparing various characteristics of these extracted functional graphs and relating them back to the branching ratio and complexity of the same networks. This approach provides the foundations for understanding how functional connectivity structure can support various dynamical regimes in networks of neurons *in vitro*.

### 2.1. Experimental details

#### 2.1.1. *In vitro* neuronal network preparation

**Figure 1:**
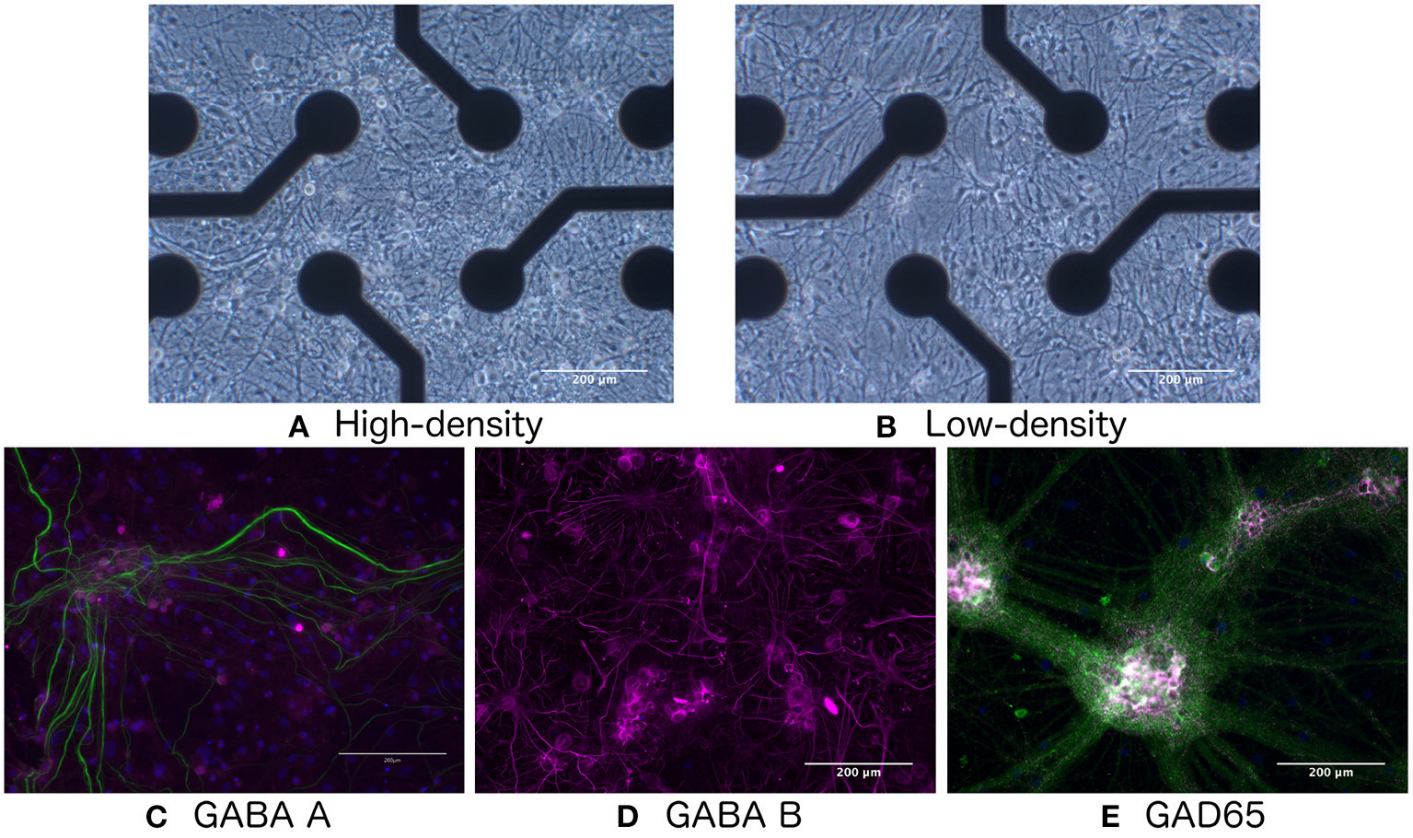
Bright-field images of **(A)** high- and **(B)** low-density networks. Fluorescence images showing the presence of **(C)** GABA-A, **(D)** GABA-B, and **(E)** GAD65. Green indicates neurofilament heavy.

Rat cortical neurons (A1084001, Thermo-Fisher Scientific) were seeded on Cytoview 6-well plates coated with polyethyleneimine (Polysciences) and natural mouse laminin (Thermo Fisher Scientific) at a density of 900 cells/mm^2^ (low-density, 95,000 cells/well, *N* = 3 wells, hereafter referred to as LD1–LD3) and 1,800 cells/mm^2^ (high-density, 187,000 cells/well, *N* = 3 wells, HD1–HD3), as shown in Figures 1A,B. Co-cultures with rat primary cortical astrocytes (N7745-100, Thermo Fisher Scientific) were established through concurrent seeding at 90 and 180 cells/mm^2^ for low- and high-density cultures, respectively. All cultures were maintained with medium consisting of 95% Neurobasal Plus, 2% B27 Plus Supplement (50X), 1% GlutaMax Supplement, and 2% PenStrep (all from Thermo Fisher Scientific). Half of the culture media was changed once per week during the course of the experiment, except in the first week *in vitro*, when media change was performed three times during the week, and immediately following chemical perturbation (see Section 2.1.2), when the media was exchanged twice. During seeding, rock inhibitor (Y-27632, Sigma Aldrich) was added to the medium.

#### 2.1.2. Electrophysiology

A CytoView MEA plate (M384-tMEA-6B, Axion Biosystems) was used for all *in vitro* electrophysiology recordings. Each well of the MEA plate consists of 64 PEDOT electrodes (100 μm diameter, 300 μm inter-electrode spacing) arranged in an 8 × 8 grid at the center of each well. Daily recordings were performed with a Maestro Pro (Axion Biosystems) at the same time each day, from days *in vitro* (DIV) 25–49. The cultures were allowed to equilibrate for at least 15 min prior to each recording and incubated during the recording to ensure stable activity.

Activity was captured using AxIS 2.4 software (Axion Biosystems). Spike detection was performed on the raw data with the same software using the adaptive threshold method with a threshold of ±7 standard deviations from the median of the signal.

For the excitation-to-inhibition ratio (E/I) disruption assay, γ-aminobutyric acid (GABA; A5835, Sigma-Aldrich) was diluted in DPBS^−/−^ and added immediately preceding recording on DIV 50.

#### 2.1.3. Immunocytochemistry

For immunocytochemistry, an IBIDI 8-well (80841, IBIDI) was coated with poly-L-ornithine (Sigma Aldrich) and natural mouse laminin (Thermo Fisher Scientific) and seeded with rat cortical neurons and astrocytes at a density of 650 and 50 cells/mm^2^, respectively. Neurobasal medium was exchanged 50% every other day until fixing. All cultures were kept in humidified incubator (37°C, 5% CO_2_).

For imaging, cells were fixed with 4% paraformaldehyde for 15 min before washing with D-PBS^−/−^, followed by 2 h blocking with 5% goat serum 0.3% Triton X in D-PBS^−/−^. Primary antibodies (neurofilament heavy (ab8135) at 1:1,000, GAD65 (ab26113) at 1:500, GABA-A (ab94585) at 1:100, GABA-B (ab55051) at 1:100, all primary antibodies from Abcam) were diluted in 1% goat serum, 0.1% Triton X in D-PBS^−/−^ and left overnight at 4°C. The following day, primary antibodies were removed and the cells washed prior to staining with secondary antibodies. Secondary antibodies (goat-anti-rabbit 647 Alexa Fluor, goat-anti-mouse 488 Alexa Fluor, Thermo Fischer Scientific) were added in 1% goat serum, 0.1% Triton X in D-PBS^−/−^ at a dilution of 1:1,000 for 2 h. Hoechst was added during the last 5 min of secondary incubation at a dilution of 1:5,000. The cells were mounted and left at 4°C overnight before microscopy. Fluorescence microscopy was performed with an EVOS Microscope M5000 Imaging System, while live cell imaging was performed with Zeiss Axio Vert. 25. All images were processed using Fiji software.

As shown in Figures 1C–E, immunocytochemistry results confirmed the presence of GABA-A and GABA-B receptors, as well as the GAD65 enzyme produced by GABA-ergic interneurons, indicating that the network would be responsive to E/I disruption by the addition of GABA.

### 2.2. Neuronal avalanche dynamics

The dynamics of the network activity were considered by evaluating the size and duration distributions of neuronal avalanches, the branching ratio, and a measure of the complexity. The Neural Complexity and Criticality (NCC) Toolbox was used to perform power law fitting, compute the complexity, and obtain the conventional estimate of the branching ratio (Marshall et al., 2016). The multiple regression (MR) estimator proposed by Wilting and Priesemann (2018) was also used to estimate the branching ratio to take into consideration the effect of subsampling.

#### 2.2.1. Avalanche detection

**Figure 2:**
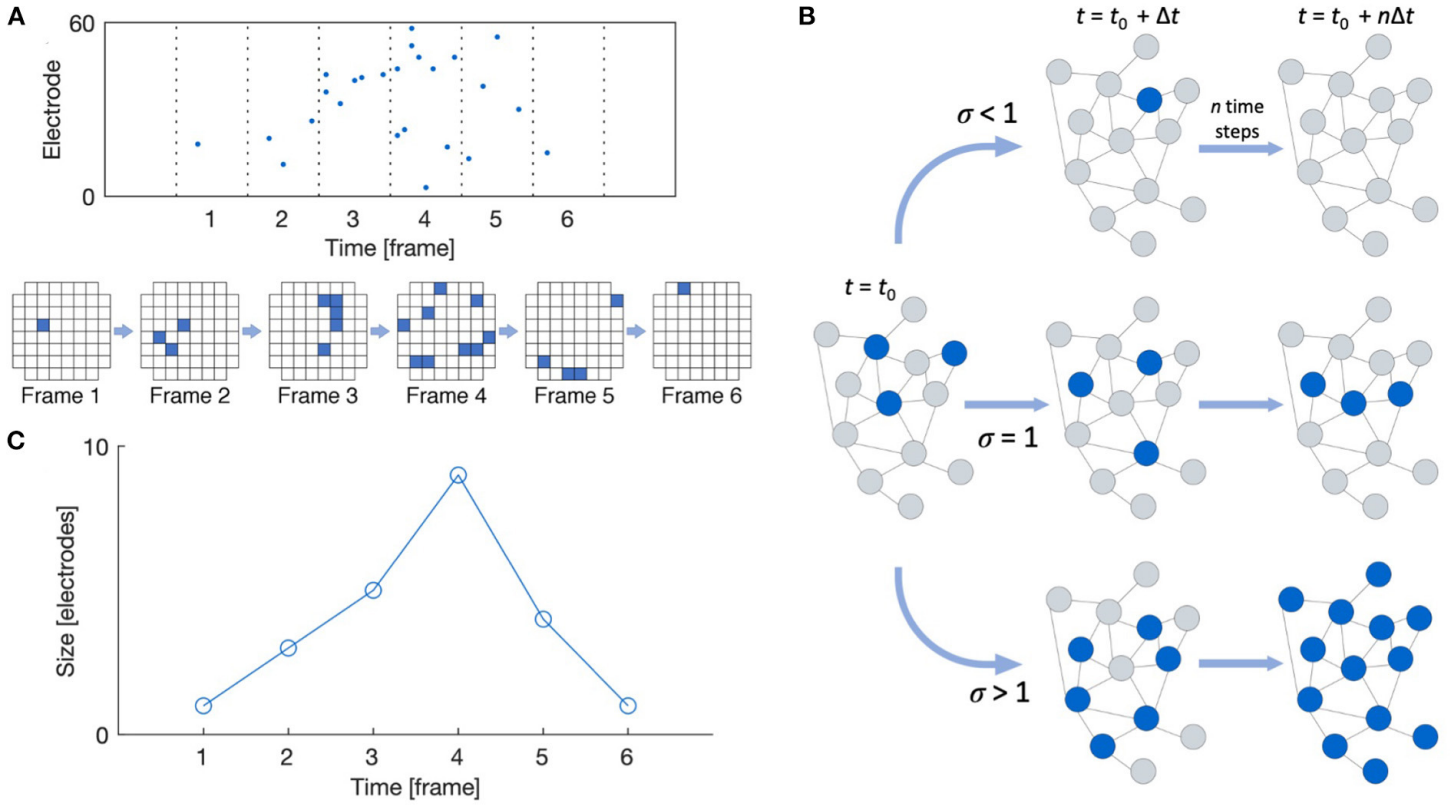
Neuronal avalanche definitions. **(A)** Raster plot and schematic showing a typical MEA layout with active recording channels shown in blue. A neuronal avalanche is defined as a sequence of active time frames bounded before and after by empty frames. **(B)** The branching ratio is defined as the ratio of descendants to ancestors. **(C)** The avalanche shape is defined as the size of the avalanche as a function of time. Reproduced from Heiney et al. (2021).

Neuronal avalanches were detected following the work by Beggs and Plenz (2003) and Pasquale et al. (2008), as shown in Figure 2A. Recordings were divided into time bins of width Δ*t*, and a time bin was considered active if any of the recording channels showed spiking activity in that bin. Avalanches were then detected as sequences of active time bins bounded before and after by time bins with no activity. The size of an avalanche can be defined in one of two ways: (1) the number of active recording channels during the avalanche, or (2) the number of spikes that occur during the avalanche; we here consider the former definition. The duration of an avalanche is defined as the number of active time bins spanning the avalanche. Multiple time bin widths (Δ*t* = 1, 2, 4, 8, 12, 16, 24, and 32 ms) were considered when performing spike binning for avalanche detection.

#### 2.2.2. Size and duration distributions

The probability distributions of the avalanche size and duration were then computed. In a network in the critical state, these distributions should follow power laws of the form


(1) $$\begin{array}{c} P\left(S\right)\propto S^{-\alpha },\\ P\left(D\right)\propto D^{-\beta },\\ \langle S\rangle \left(D\right)\propto D^{1/\sigma \nu z}, \end{array}$$

where *P*(·) is the probability distribution; *S* is the avalanche size; *D* is the avalanche duration; 〈*S*〉(*D*) is the expectation value of the avalanche size given its duration; and α, β, and 1/σ*νz* are the characteristic critical exponents of the system. For a network in the critical state, 1/σ*νz* = (α − 1)/(β − 1). Networks in the sub- and supercritical states exhibit exponential and bimodal distributions, respectively.

Using the NCC Toolbox (Marshall et al., 2016), power laws of the form given in Equation (1) were fit to the empirical distributions using maximum likelihood estimation (MLE). In the method in this toolbox, truncated power laws are fit to the largest range of size or duration values that yield a significant fit, and the goodness of fit is computed using the method outlined by Clauset et al. (2009).

#### 2.2.3. Avalanche shape

The shape of the detected avalanches was also evaluated using the NCC Toolbox (Marshall et al., 2016). The shape of an avalanche is defined as its size over each time frame of its duration, as shown in Figure 2C. Raw avalanche shape profiles *s*(*t*) can be compared by rescaling their size and duration according to the scaling function *s*(*t, D*) ∝ *D*^γ^*F*(*t*/*D*) (Friedman et al., 2012). For systems in the critical state, the scaled shapes *s*(*t, D*)*D*^−γ^ plotted against the scaled time *t*/*D* should collapse onto the same profile *F*(*t*/*D*). Additionally, this scaling exponent γ should relate to the exponent from Equation (1) as γ = 1/σ*νz* − 1 for critical systems.

#### 2.2.4. Branching ratio

As shown in Figure 2, the branching ratio *m* is defined as the mean ratio of the number of “descendant” nodes (active recording channels here) in time bin *t* + 1 to the number of “ancestor” nodes in time bin *t*. In the conventional estimate, descendant nodes are simply taken as the observed nodes that are active in the time bin following the activity of the ancestor nodes, and the mean is taken such that a “descendant” time bin may contain zero activity but an “ancestor” may not (Haldeman and Beggs, 2005). However, this approach does not yield an accurate estimate in the face of subsampling.

The MR estimator method was developed to take into consideration how subsampling greatly biases the estimate of the branching ratio (Wilting and Priesemann, 2018). When recording activity from neuronal networks, only a small percentage of the network is being observed; in the present experiment, each network contained approximately 100,000–200,000 cells but only at most 64 active recording sites. This subsampling obfuscates how activity spreads over the network, but the MR estimator is able to give an accurate estimate of the branching ratio even under strong subsampling.

In the MR estimator method, rather than consider the biased regression of the activity between the activity at *t* and that at *t* + 1, multiple regressions *r*_*k*_ are taken between times *t* and *t* + *k* for many lags *k*. Under subsampling, these regressions *r*_*k*_ are all biased by the same factor *b*, as $r_{k}=bm^{k}$, where *m* is the true branching ratio. Thus, an estimate of the branching ratio $\hat{m}$ can be calculated by obtaining multiple regressions and fitting them to an exponential model (Wilting and Priesemann, 2018).

#### 2.2.5. Complexity

The complexity of the network activity was also computed using the NCC Toolbox (Marshall et al., 2016), which uses the metric developed by Tononi et al. (1994) modified to correct for subsampling biases. This measure of complexity is based on the entropy, which is given for a system of neurons as


(2) $$\begin{array}{l} H\left(X\right)=-\sum p\left(x_{i}\right)\text{log}\left(p\left(x_{i}\right)\right), \end{array}$$

where *x*_*i*_ is the joint state of all neurons and *p*(*x*_*i*_) is the probability of that state occurring.

From this, the integration $I\left(X_{j}^{k}\right)$ of the *j*th unique set of *k* neurons, $X_{j}^{k}$, is computed as


(3) $$\begin{array}{l} I\left(X_{j}^{k}\right)=\left(\underset{j^{\prime }\in k}{\sum }H\left(X_{j^{\prime }}^{1}\right)\right)-H\left(X_{j}^{k}\right). \end{array}$$

The integration formulated in this way describes the degree of coordination of the activity of the set of neurons $X_{j}^{k}$.

The complexity of a system is low when all neurons are coordinated but also when their activity is completely unrelated, so the complexity measure should be high at intermediate values of *I*. The complexity proposed by Tononi et al. (1994) is given by


(4) $$\begin{array}{l} c\left(X\right)=\frac{1}{N}\overset{N}{\underset{k=2}{\sum }}\left[\left(\frac{k-1}{N-1}\right)I\left(X\right)-{\langle I\left(X_{j}^{k}\right)\rangle }_{j}\right]. \end{array}$$

For more details on the computation and a graphical interpretation of this measure, see the NCC Toolbox documentation (Marshall et al., 2016).

### 2.3. Functional connectivity

To evaluate the relationship between the avalanche dynamics observed in the networks and their toplogies, graphs of the functional connectivity were obtained from the data and analyzed. This section outlines the methods of extracting the functional connectivity and characterizing the obtained graphs.

#### 2.3.1. Generation of functional connectivity graphs

Weighted functional connectivity graphs were extracted from the spiking data as follows using the cross-correlation between pairs of spike trains. Spike trains were obtained for every recording channel by binning the spiking data with the same time bin widths used for the avalanche detection (Δ*t* = 1, 2, 4, 8, 12, 16, 24, and 32 ms). The cross-correlation was taken between all pairs of spike trains with a maximum lag of 100 ms. The weight of the connection between two nodes was obtained by first calculating the normalized correlation *R*_*xy*_ between their binned spike trains *x* and *y*, as


(5) $$\begin{array}{l} R_{xy}\left(\tau \right)=\frac{1}{N_{x}N_{y}}\overset{N_{x}}{\underset{s=1}{\sum }}x\left(t_{s}\right)y\left(t_{s}-\tau \right), \end{array}$$

where *N*_*x*_ and *N*_*y*_ are the numbers of spikes in the binned spike trains *x* and *y*, respectively; *t*_*s*_ is the time bin containing spike *s* in the spike train of *x*; and τ is the lag. The adjacency matrix describing the weights *w*_*ij*_ of all connections was then obtained by averaging the cross-correlation values *R*_*xy*_ up to a maximum lag of 100 ms.

To eliminate spurious connections, any correlations with *p* < 0.1 were considered insignificant and removed, and any edges with weights below a hard threshold of *R* = 0.1 were removed. Although it is a common approach to set the threshold based on the correlations found for corresponding shuffled spike trains, we chose to use a hard threshold for all networks to avoid enhancing any between-group differences stemming from the disparate thresholds that would arise from this approach (van den Heuvel et al., 2017; Hallquist and Hillary, 2018). Additionally, the relatively high threshold was used instead of the more common 0.05 to reduce the number of false positive or spurious connections in the networks and better approximate the desired 2FN–1FP ratio discussed by Zalesky et al. (2016); however, using thresholds of 0.05 and 0.02 for graph extraction were found to yield similar results to the selected threshold of 0.1.

The corresponding binary graphs were generated by setting all non-zero weights to 1, and the analysis described in the following section was also performed on the binary metrics to evaluate the consistency in the results.

#### 2.3.2. Graph analysis

The following metrics were calculated for each of the functional connectivity graphs: clustering coefficient *C*, characteristic path length *L*, network diameter *D*, maximum degree *k*_max_, and hub count *N*_hub_. This section briefly describes how each of these is computed.

The clustering coefficient *C* is defined as the fraction of a node's neighbors that are connected to another of that node's neighbors, as


(6) $$\begin{array}{l} C=\frac{1}{N}\underset{i}{\sum }c_{i}=\frac{1}{N}\underset{i}{\sum }\frac{1}{k_{i}\left(k_{i}-1\right)}\underset{j,k}{\sum }{\left({ŵ}_{ij}{ŵ}_{jk}{ŵ}_{ik}\right)}^{1/3}, \end{array}$$

where the local coefficient *c*_*i*_ of each node is defined using the method by Onnela et al. (2005) for weighted graphs, with $\hat{w_{ij}}=w_{ij}/\text{max}\left(w\right)$ and *k*_*i*_ the number of edges connected to node *i*.

The characteristic path length *L* is defined as the average of the shortest path length between all possible pairs of nodes, as


(7) $$\begin{array}{l} L=\frac{1}{N\left(N-1\right)}\underset{i\neq j}{\sum }d_{ij}, \end{array}$$

where *d*_*ij*_ is the shortest distance between nodes *i* and *j*. The distance between a pair of adjacent nodes *x* and *y* connected by an edge with weight *w*_*xy*_ is defined as *d*_*xy*_ = 1/*w*_*xy*_. The network diameter *D* is then defined as the longest of these shortest path lengths between all pairs of nodes, that is, the shortest possible path between the two most distant nodes.

The degree *k* of a node in a weighted graph is defined as the sum of the weights of the node's edges, and the maximum degree *k*_max_ is the highest degree occurring in a graph. The number of hubs is then defined as the number of nodes with degree exceeding one standard deviation above the mean degree of the network.

#### 2.3.3. Normalization of graph metrics with small-world null models

Unsurprisingly, the extracted graphs had different numbers of nodes and mean degrees, and because of this, many of the graph metrics described in the previous section cannot be directly compared (van Wijk et al., 2010; Hallquist and Hillary, 2018). To enable comparison of all of the graphs, the metrics were normalized by generating 200 null models, calculating the corresponding metrics from those models, and normalizing the empirical values with respect to the mean of the corresponding value across the null models.

For the null model, a small-world model with the same number of nodes, mean degree, and sparsity as the empirical graph was selected, where sparsity is defined as the ratio of the number of existing edges in the network to the number of possible edges, i.e., edges in a fully connected network of the same size. These were created by first generating binary small-world graphs using the Watts–Strogatz model with β = 0.02 (Watts and Strogatz, 1998). All nonzero elements in the adjacency matrix were then replaced by the weights from the empirical adjacency matrix, retaining the order in which they occur in the empirical model.

This null model was selected because the majority of the extracted functional graphs were small-world, as evaluated using the small-world propensity, which will be described below. Specifically, for the high- and low-density networks, 68.7 and 61.7% of the graphs were small-world for the high- and low-density networks, respectively, and their respective mean SWPs were 0.72 and 0.61. These percentages and means were taken across all considered DIVs and time bin widths for each density.

The SWP ϕ relies on the characteristic path length *L* and the clustering coefficient *C*, which are both defined in the previous section. The SWP is then defined as Muldoon et al. (2016)


(8) $$\begin{array}{c} \phi =1-\sqrt{\frac{\Delta_{C}^{2}+\Delta_{L}^{2}}{2}},\\ \Delta_{C}=\frac{C_{latt}-C_{obs}}{C_{latt}-C_{rand}},\\ \Delta_{L}=\frac{L_{obs}-L_{rand}}{L_{latt}-L_{rand}}. \end{array}$$

This metric introduces the ratios Δ_*C*_ and Δ_*L*_ to represent the fractional deviation of the observed graph from the corresponding null case, i.e., a lattice graph for the clustering coefficient and a random graph for the characteristic path length. On the basis of this metric, a graph is said to be small-world if ϕ > 0.6.

### 2.4. Correlations between avalanche and graph metrics

To assess the relationship between the avalanche dynamics occurring on the networks and the topological structures of the functional graphs, the correlation between pairs of avalanche and graph metrics was evaluated. For this, the partial Spearman correlation was used to eliminate the effects of confounding variables. The following confounding variables were considered: the numbers of nodes and edges in the graphs, the DIV, and the number of days since the last media change.

## 3. Overview of network activity

**Figure 3:**
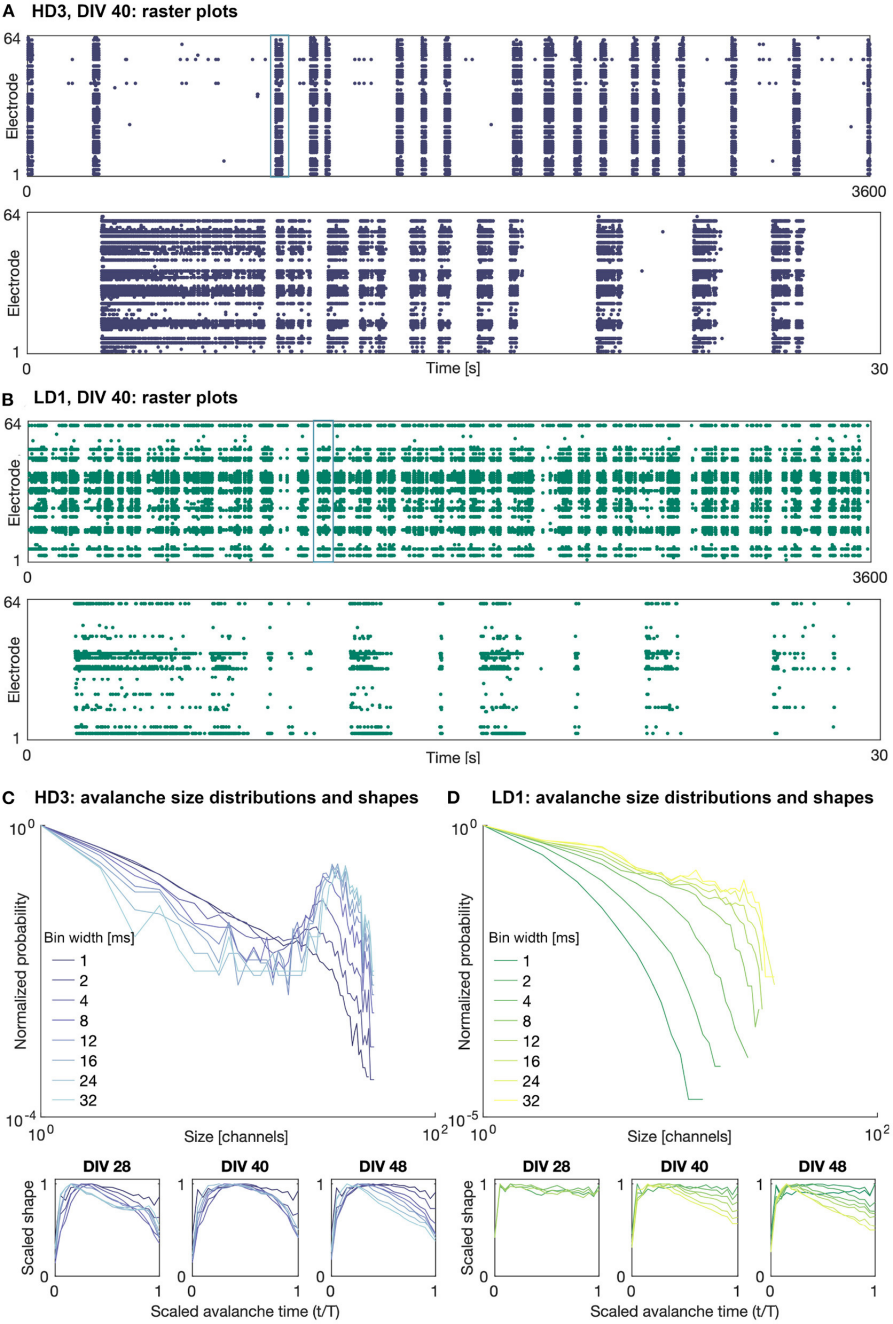
Raster plots and avalanche size distributions on DIV 40. Raster plots of the entire 1 h recording period (upper) and indicated 30 s period (lower) for **(A)** HD3 and **(B)** LD1. Avalanche size distributions and mean scaled shapes obtained with different time bin widths for **(C)** HD3 and **(D)** LD1. Distributions are normalized by the probability of size 1.

Figures 3A,B shows example raster plots of the high- and low-density network activity on DIV 40. The upper plots show the activity over the entire observation duration (1 h), and the lower plots show 30 s of activity, the timing of which is indicated in the upper plots. As shown in this figure, the activity of both the high- and low-density networks was dominated by large bursts characterized a range of time scales. The duration and frequency of these bursts varied network to network and day to day.

It should be noted that not all recording channels in the MEA were active during every observation day. The mean numbers of active recording channels for the high- and low-density networks across all recording days were approximately 50 channels (minimum: 33, maximum: 58) and 28 channels (minimum: 17, maximum: 41), respectively, where a channel is considered active if it has recorded at least 10 events. The widely variable number of active channels across densities and DIVs introduces some challenges in comparing their corresponding computational measures.

**Figure 4:**
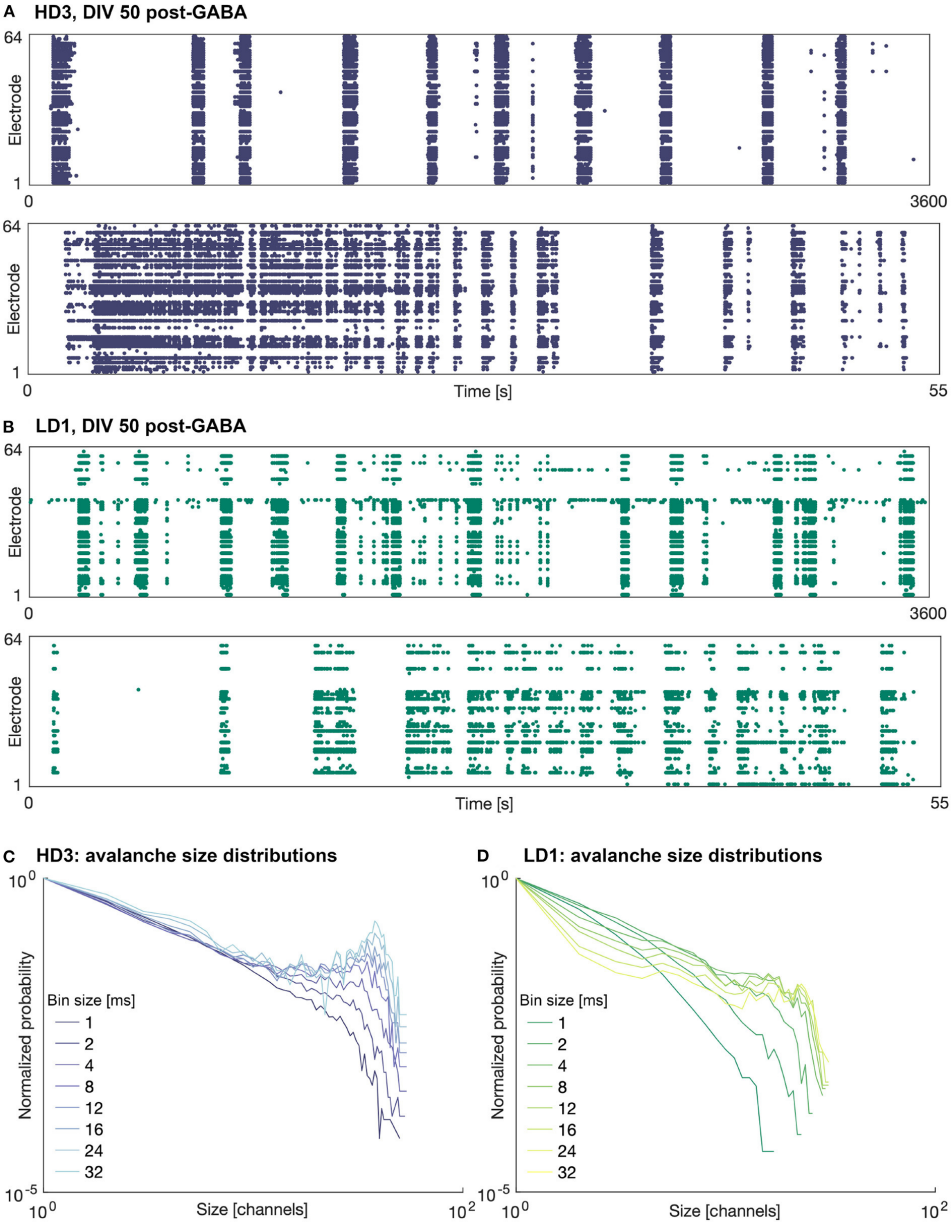
Raster plots and avalanche size distributions on DIV 50 following perturbation by GABA. Raster plots of the entire 1 h recording period (upper) and indicated 55 s period (lower) for **(A)** HD3 and **(B)** LD1. Avalanche size distributions obtained with different time bin widths normalized by the probability of size 1 for **(C)** HD3 and **(D)** LD1.

The addition of 25 μM GABA on DIV 50 reduced the amount of activity (mean firing rate) in all of the networks, as expected. In some of the networks, the activity was completely silenced; in particular one of the high-density networks (HD1) and two of the low-density networks (LD2 and LD3) did not produce any activity during the hour following perturbation with GABA. Interestingly, in those networks that continued to produce activity following perturbation, the spatiotemporal structure of the activity was not markedly different from that before perturbation—activity remained fairly synchronized and continued to be dominated by large bursts. Furthermore, the duration of these bursts tended to be greater than those prior to the perturbation, as shown in Figure 4—note the timescale of 55 s zoomed in on singular burst patterns, as compared to that of 30 s in Figure 3. The avalanche size continued to show bimodal distributions; however, in the case of HD3, the peaks at larger avalanche sizes were not as pronounced as they were prior to perturbation.

The present results contrast our previous results (Heiney et al., 2019), where perturbation with GABA was able to break the synchrony of the network activity and allow the networks to produce more complex patterns of activity. This suggests that sensitivity to GABA may vary from network to network and is likely dependent on more factors than the seeding density and level of maturation.

## 4. Neuronal avalanche dynamics

The dynamics of the *in vitro* networks were first investigated by analyzing the behavior of their neuronal avalanches. This section first presents a brief overview of the size distributions of the neuronal avalanches, which were generally found to be bimodal across a range of time bins. Then, the branching ratio and complexity results are presented. The branching ratio indicated that the networks were generally subcritical, with many in the slightly subcritical range (*m* ≈ 0.99). The complexity of the high-density networks was much higher than that of the low-density networks. Higher complexity tended to occur at later maturation time points and correspond to branching ratios closer to 1.

### 4.1. Avalanche size distributions and shapes

Examples of the avalanche size distributions are shown in Figures 3C,D for the high-density network HD3 and the low-density network LD1, respectively, on DIV 40. The distributions are shown for all considered time bin widths, ranging from 1 to 32 ms. The size and duration distributions of the networks tended to show similar features across networks and DIVs: bimodal at larger time bin widths and exponentially decaying at smaller time bin widths. In some cases, as shown in Figure 3D, the low-density networks showed only rapidly decaying avalanche distributions, as the upper limit of the size was limited by the low number of active electrodes.

The power-law fitting results did not yield significant fits for any network on any DIV, nor after perturbation with GABA. Because of this, the different estimates of 1/σ*νz* described in Section 2.2 could not be meaningfully compared.

The mean scaled avalanche shape (i.e., size vs. time over the course of an avalanche; bottom panels in Figures 3C,D) frequently showed the characteristic roughly parabolic shape, with the size peaking near the middle of an avalanche; this can be seen at intermediate time bin widths on DIVs 40 and 48 for HD3. However, in many cases, the peak was skewed earlier in the avalanche, suggesting a more rapid spread and gradual decay in activity than what is characteristic of networks at criticality; this can be seen on DIV 48 for both HD3 and LD1, particularly at higher time bin width selections. Furthermore, at larger time bin widths (>8 ms), some mean shapes were bimodal, indicating multiple cascades of activity were combined into a single detected avalanche. This was characteristic of activity in all three of the high-density networks at earlier DIVs, as shown on DIV 28 for HD3 with time bin widths of 24 and 32 ms. This suggests that the physiologically relevant timescale for these networks was in the range of 1–8 ms; given the interelectrode spacing of 300 μm in this study, these timescales are in agreement with previous reports of propagation speeds *in vitro* ranging from 30 to 300 mm/s (Jacobi and Moses, 2007).

### 4.2. Branching ratio

**Figure 5:**
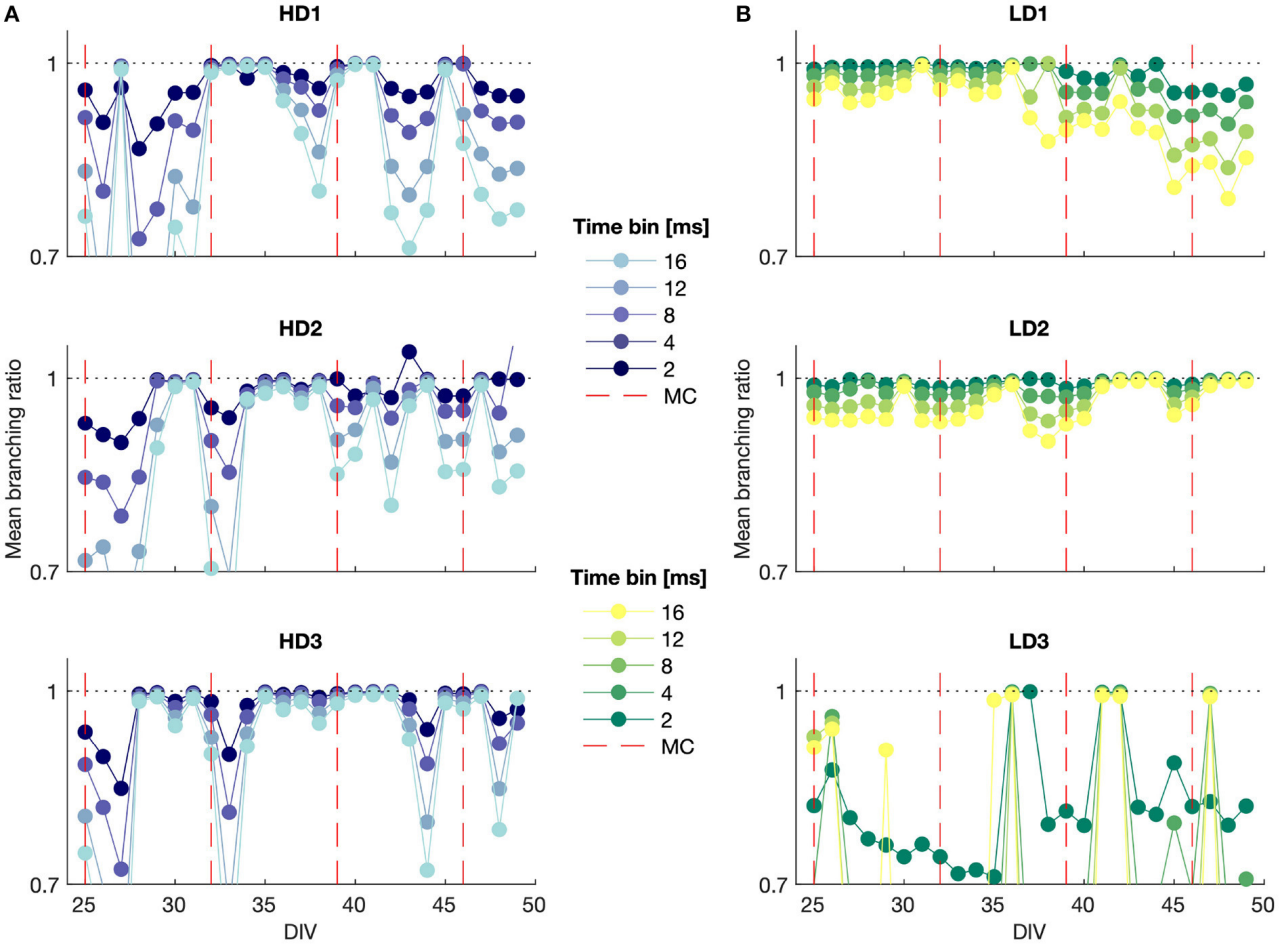
MR estimate of the branching ratio calculated for different time bin sizes plotted against DIV for **(A)** high- and **(B)** low-density networks. MC, media change.

The mean branching ratio results for each of the networks are shown in Figure 5 for mid-range time bin widths (Δ*t* = 2, 4, 8, 12, and 16 ms). The branching ratio estimate shown here was obtained with the MR estimation method to account for subsampling. A range of time bin widths was used to take into account that systems near criticality should show branching ratios near 1 regardless of time bin selection.

The branching ratio was almost always below 1, indicating a subcritical state, and it was frequently very close to 1 (*m* ≈ 0.99), representing the slightly subcritical regime (Priesemann et al., 2014). The branching ratio fell in the range 0.98 ≤ *m* < 1 in 35%, 34%, and 53% of the observations for the high-density networks and 32%, 53%, and 18% of the observations for the low-density networks. This suggests that it is common for networks to self-organize into the slightly subcritical regime *in vitro*, though deviations also occurred with some regularity.

In particular, HD3 and LD2 appeared to remain close to criticality for much of the observation time, whereas the branching ratio results for LD3 indicated consistent subcriticality. The high-density networks tended to show more drastic deviations into the subcritical regime from day to day, whereas LD1 and LD2 gradually moved, respectively, away from and toward the slightly subcritical regime over time.

It is noteworthy that the subsampling-corrected branching ratio estimate indicated a consistent subcritical or slightly subcritical state for each of the networks over all observation days, whereas the avalanche size consistently showed a bimodal distribution (Figures 3C,D), which is indicative of supercriticality, particularly in the high-density networks for time bin widths of 4 ms and higher. These two observations being at odds suggests either that bimodal size distributions are not a definitive indicator of supercritical behavior when considering strongly subsampled *in vitro* activity, or that the MR estimate of the branching ratio does not accurately capture the branching activity of networks with branching ratios exceeding 1.

The conventional estimate of the branching ratio (results not shown) yielded interesting cyclic behavior at the smallest time bin width (Δ*t* = 1 ms) that aligned with the dates on which the culture medium was changed. This suggests that information propagation in the network may be sensitive to the availability of metabolic resources and the presence of factors released by the cells in the culture. The days on which the media were changed are shown in the MR branching ratio estimate results in Figure 5; however, while some of these time points correspond to shifts to a more subcritical state immediately preceding or following media change as indicated by this branching ratio estimate, the media change did not affect the MR estimate of the branching ratio in as consistent a manner as it did the conventional estimate.

### 4.3. Complexity

**Figure 6:**
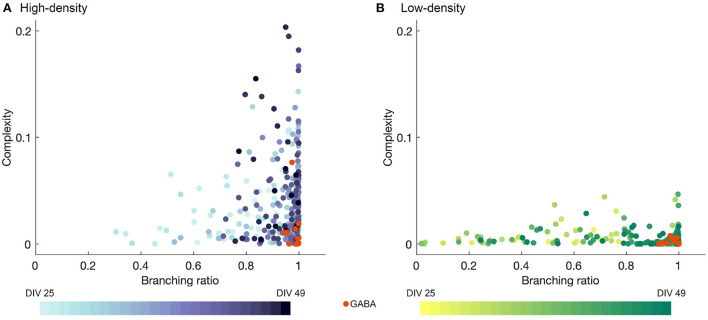
Complexity plotted against branching ratio for **(A)** high- and **(B)** low-density networks, obtained for mid-range time bin sizes (Δ*t* = 2, 4, 8, 12, and 16 ms). Darker colors represent later DIVs, and the orange data points represent the activity after the addition of GABA. In **(A)**, two outliers with branching ratios exceeding 1 (*m* = 1.05 and 1.08) were removed for the sake of visualization.

The complexity is plotted against the branching ratio in Figures 6A,B for the high- and low-density networks. Each data point represents a single observation day with the values computed for a single time bin width, with intermediate time bin widths included (Δ*t* = 2, 4, 8, 12, and 16 ms), as in Figure 5 and Section 4.2. The colors represent the different observation DIVs, with darker colors corresponding to later time points in the observation period, and the orange points represent the results after perturbation with GABA.

As shown in Figure 6, the high-density networks tended to have much higher complexity values than the low-density networks (*p* < 1 × 10^−50^, two-sample T-test), with the peak complexity in the high-density case reaching approximately four times that in the low-density case. The fluctuations in the complexity day by day for each bin width selection were quite erratic, but the higher complexity results tended to occur at later DIVs, especially for the high-density networks, as indicated by the darker color of the higher points in Figure 6A. Higher complexity values also tended to be accompanied by branching ratios closer to 1, though the converse was not always true.

Perturbation with GABA tended to result in low complexity values, while not greatly disrupting the branching ratio. However, the drastically reduced level of activity in many of the networks may affect the accuracy of the estimates of the branching ratio.

## 5. Functional graph characteristics

**Figure 7:**
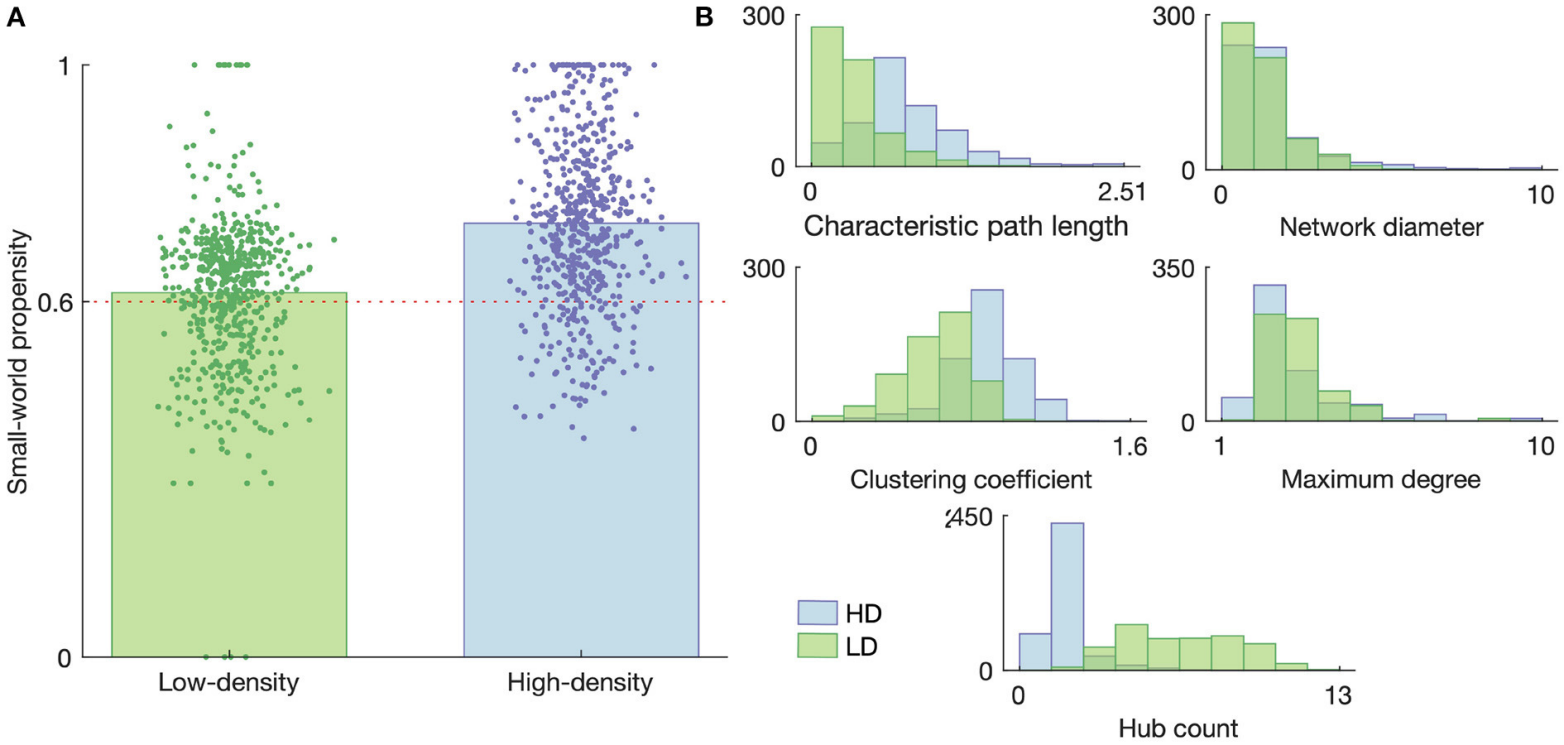
Graph properties of the high- and low-density networks. **(A)** Small-world propensity. Each data point represents a functional connectivity graph obtained from network activity on a given day with a given time bin size; they are given random horizontal jitters for the sake of visualization. The heights of the bars represent the mean small-world propensity across all such graphs. The horizontal dashed line represents the threshold above which a graph is considered “small-world.” **(B)** Histograms of the small-world-normalized graph metrics considered in this study. Histograms represent graphs obtained on all DIVs with all considered time bin sizes.

A summary of the graph characteristics is shown in Figure 7, with Figure 7A showing the small-worldness of each obtained graph and Figure 7B showing the distributions of each of the considered small-world-normalized graph measures for the low- and high-density networks. The results in Figure 7A demonstrate that the majority of both the low- and high-density networks tend to organize into a small-world topology, though the high-density networks are generally more small-world than the low-density networks (*p* < 1 × 10^−36^, two-sample *t*-test). This result motivated the use of small-world null models for graph metric normalization, rather than using randomly connected null models or non-normalized metrics.

As shown in Figure 7B, many of the normalized network metrics tended to show somewhat different distributions for the low- and high-density networks. The path length and network diameter tended to be larger for the high-density networks than the low-density networks, suggesting a greater separation between nodes. This greater separation is also consistent with the lower hub count (number of nodes of degree greater than the mean degree) in the high-density networks, as fewer hubs would generally lead to less connection between disparate parts of the network.

It is also interesting to note that the normalized characteristic path length always fell below 1 for both networks, meaning the empirical networks consistently had lower path lengths then their small-world null counterparts. A similar trend in the hub count, excluding a few dozen outliers, along with a consistently high maximum degree, suggests the path length was reduced relative to the small-world case by the presence of a few very highly connected nodes rather than a large number of less-connected nodes.

### 5.1. Relating functional graphs to avalanche dynamics

To evaluate the relationship between the characteristics of the functional graphs obtained from the *in vitro* networks and their avalanche dynamics, the partial correlation was evaluated, as described in Section 2.4. The metrics used to describe and compare the graphs were those described in Section 2.3.2—the clustering coefficient *C*, the characteristic path length *L*, the network diameter *D*, the maximum degree *k*_max_, and the hub count *N*_hub_—and they were normalized with respect to small-world null models, as described in Section 2.3.3.

Figure 7 demonstrates that the majority of the functional connectivity graphs could be considered small-world, justifying the normalization by small-world null models to compare networks of different sizes.

The correlation results are listed in Table 1. As shown in the table, although significant, the correlations are fairly weak. This is likely due to the small number of nodes in our extracted graphs; graph metrics are notoriously challenging to calculate for small graphs (van Wijk et al., 2010). It should be noted that the results obtained for the corresponding binary graphs were fairly consistent with the weighted graph results. In two instances, significant correlations were found in the binary case and not in the weighted case; for these, the correlation from the binary case is reported in the table and denoted by an asterisk.

**Table 1:** Partial correlation of graph metrics with branching ratio and complexity.

	***C***	***L***	***D***	***k*_max_**	***N*_hub_**
**HD**					
*m*	NS	0.12	NS	−0.17	NS
*c*	0.11^*^	−0.42	−0.40	0.37	0.22
**LD**					
*m*	0.20	0.14	0.10	−0.35	−0.11
*c*	−0.14	NS	−0.11	NS	NS

* Correlation reported for binary graph (NS for weighted); HD, high-density networks; LD, low-density networks; NS, not significant; *m*, branching ratio; *c*, complexity; *C*, clustering coefficient; *L*, characteristic path length; *D*, network diameter; *k*_max_, maximum degree; *N*_hub_, number of hubs.

### 5.2. Branching ratio and graph features

The branching ratio showed a positive correlation with the characteristic path length and the network diameter, with the correlation with path length arising in both the high- and low-density networks. This suggests that networks are better able to sustain activity when their nodes are more functionally distant from one another. In the present case, larger branching ratios were those that approached *m* = 1 most closely (see Figure 5), meaning larger path lengths also correspond to cases where the networks are closer to the critical point.

Similarly, higher branching ratios also showed correspondence with lower maximum degrees and a greater number of hubs (nodes with degree exceeding one standard deviation above the mean degree). This is consistent with the path length observations, as increasing the maximum degree would reduce the distance between nodes, and reducing the maximum degree can also reduce the mean and therefore may increase the number of hubs. The clustering coefficient also showed a positive correlation with the branching ratio in the low-density networks.

Taken together, these results suggest that activity can be better sustained in networks with functionally distant nodes that tend to cluster together, without any major hubs in the network. However, crucially, because the networks here are drastically subsampled to obtain the corresponding functional graphs, it is certainly possible that there exist hubs outside of the observation zone and our analysis does not capture the contribution of these hubs to the activity. Despite this, it is interesting that when high-degree hubs *are* observed, these cases tend to also have a lower branching ratio, suggestive of networks further from criticality.

If a network of purely excitatory nodes is considered, the above correlations are generally opposite what one would expect; that is, more highly connected nodes and shorter paths between nodes would be expected to give rise to more propagated activity and thus a higher branching ratio. That a greater degree of integration in the functional graphs would correspond to reduced propagation suggests that the more highly connected networks come along with higher levels of inhibition, halting the spread of activity early in an avalanche.

### 5.3. Complexity and graph features

The correlations between the complexity and each of the graph features tended to be significant only for the high-density networks, and so the focus of the discussion here will be for these networks. The lack of significant correlations for the low-density networks is likely due to the very little network-to-network variability in their complexity (see Figure 6B).

The path length and network diameter were negatively correlated with the complexity, indicating networks with closer functional connection between pairs of nodes tended to produce more complex activity. This suggests that greater integration is needed to sustain complex activity, and a greater path length likely reduces complexity by segregating the network and lessening the coordination among nodes. The complexity was also positively correlated with the maximum degree and hub count, and weakly with the clustering coefficient. This again suggests that a greater level of integration in the network contributes to more complex activity.

The complexity correlations are more challenging to definitively interpret than the branching ratio correlations. As described in Section 2.2.5, the complexity is high when a balance is struck between integration and segregation—when nodes act together and allow activity to spread but not when high synchrony is exhibited in the network. Thus, when the complexity is low, it may be due to repetitive or non-propagating activity, or to the network being overwhelmed. However, given the trends described above and that the branching ratio estimate tended to remain below 1, low complexity values in the present study likely resulted from a network being too functionally segregated, rather than too integrated. The results here then together indicate that the high-density networks showed greater complexity as they became more integrated.

## 6. Discussion

Although there is evidence that networks of neurons may tend to self-organize to the critical state (Beggs and Plenz, 2003; Tetzlaff et al., 2010) or the slightly subcritical regime (Priesemann et al., 2014) to optimize information processing (Shew et al., 2009, 2011), not all *in vitro* networks reach criticality during their maturation (Pasquale et al., 2008). In the present study, none of the six networks, prepared with two different plating densities, showed evidence of criticality after maturing for 50 DIVs. However, their behavior still provided some insight into the dynamics of their activity and how it can be related to features of their functional connectivity. This study demonstrates that analytical approaches of criticality and connectivity are complementary and can provide greater insight into network dynamics than either approach alone.

The branching ratio results indicate that the high-density networks tended to maintain subcritical dynamics closer to criticality (branching ratio closer to 1) than the low-density networks, though their presumed higher metabolic activity also may have made them more susceptible to variation between media changes (see Figure 5); media changes would also have produced variations in neurotransmitter concentrations. However, it should be noted that the branching ratio results were at odds with the observed bimodal avalanche distributions, which are indicative of supercriticality. Thus, it is unclear if the MR estimate of the branching ratio is able to accurately capture the branching behavior of systems with branching ratios exceeding 1, or whether the appearance of bimodality in the size distribution is indeed a definitive an indicator of supercriticality. Furthermore, a branching ratio below 1 may also arise from a high probability of spontaneous activation; this causes the susceptibility (the probability of a node's activity being affected by the activity of its neighbors; maximized at criticality Williams-García et al., 2014) to peak at lower branching ratios. This flexibility in how a system may be optimized for computation underlies the concept of quasicriticality, whereby systems deviate from criticality under the influence of external stimuli, in order to maintain maximal susceptibility, but maintain the same spatiotemporal scaling relationships seen at criticality (Williams-García et al., 2014; Girardi-Schappo et al., 2020; Fosque et al., 2021). The fluctuations in branching ratio with media changes are also consistent with quasicriticality, as the resources available to the networks influence how they flexibly tune their dynamics around criticality.

The activity produced by the high-density networks was also much more complex than that of the low-density networks (see Figure 6, particularly at later DIVs). Timme et al. (2016) have noted that complexity, quantified using the same metric applied in the present study (Marshall et al., 2016), is maximized at criticality and confirmed this finding both in experimental data from dissociated hippocampal networks and a critical model. Similar findings have also been reported by Lotfi et al. (2021) for *in vivo* cortical spiking data using the Jensen disequilibrium as a measure of criticality. Although we did not see evidence of criticality in our networks, it is possible that the higher complexity value in the high-density networks, especially at later DIVs, point to the networks approaching a critical state.

The mean avalanche shape did not consistently show the parabolic trajectory expected for systems at criticality. In many cases, the shape exhibited a leftward skew (see bottom panels of Figures 3C,D), indicating that more of the activity tended to occur earlier in the course of an avalanche. This may be suggestive of the networks exhibiting high levels of excitation not well balanced by inhibition: activity at the start of an avalanche setting off a sudden cascade that overwhelms the network and quickly dies off as much of the system becomes refractory. Nandi et al. (2022) observed a similar connection between avalanche shape and E/I ratio in their simulations of avalanche shape behavior; however, in their study, the observed an increasingly rightward skew as the ratio of inhibitory neurons in the population was increased, with no leftward skew in the case of pure excitation. This evidence of high levels of excitation in our networks is also in line with our previous finding that *in vitro* networks can be pushed toward criticality by chemically manipulating the E/I ratio (Heiney et al., 2019), despite this effect not being reproduced to the same degree in the present study (see Figure 4). The importance of inhibition ratio is also echoed in the model developed by Tetzlaff et al. (2010), which relates criticality to known phases of morphological development; they found that inhibition influences a network's ability to reach criticality, with purely excitatory networks remaining in the slightly supercritical phase.

In terms of the functional connectivity features, the high-density networks were more small-world and had longer path lengths and higher clustering coefficients with slightly lower maximum degrees. These features—longer path length, higher clustering coefficient, and lower maximum degree—also correlated with networks being closer to criticality (branching ratio closer to 1) for both the high- and low-density networks. The higher clustering coefficient points to greater integration, whereas the longer path length and lower maximum degree indicate less integration, suggesting the networks are working to strike a balance in the level of integration in the network. It has been suggested that clustering may support the emergence of Griffiths phases (Moretti and Muñoz, 2013), which broadens the range of parameters in a system that can give rise to criticality, thus allowing less-precise tuning to reach a near-critical state. This occurs because clustering enhances the spread of activity at a smaller scale, among those neurons joined in a cluster, while limiting the spread between clusters at a larger scale; thus, heterogenous clusters will influence the scaling of avalanches in a way that broadens the critical regime (Moretti and Muñoz, 2013).

Along the same lines, modularity may favor the emergence of criticality; this is in line with previous theoretical findings that modularity broadens the critical regime (Rubinov et al., 2011; Wang and Zhou, 2012), which allows the fine-tuning of dynamics near and within this regime. In particular, modules in a network following these trends would be connected by many small hubs rather than few major hubs—this kind of topology would produce high characteristic path lengths while maintaining a low maximum degree and many hubs. Modularity is one way networks can strike a balance between integration and segregation and produce coordinated—but not entirely synchronous—activity, which in turn is beneficial for the propagation and modulation of information in the network. However, the small size of our networks precluded a more rigorous investigation of the modularity.

Although the findings here demonstrate the complementarity of the analytical approaches used, there were a number of limitations. First, both the dynamics and connectivity analysis would be improved by increasing the number of recording channels. Spatial subsampling is known to bias the measures used here, and network features are challenging to reliably compute on small graphs (van Wijk et al., 2010). Additionally, not observing self-organized criticality in our networks limited the comparison of different dynamical regimes, as only subcriticality could be identified from the branching ratio. Had networks been observed in more varied dynamical regimes, i.e., in critical and supercritical states with branching ratios equal to and exceeding 1, perhaps different network features would have been observed, allowing us to more definitively identify features supporting criticality. Additional analysis could also have been conducted in this case to evaluate whether there was a correspondence between the dynamical regime and the computational capacity of the network, by electrically stimulating the networks and observing the response following stimulation, as has been performed previously in slice cultures (Shew et al., 2009, 2011).

Furthermore, including a greater number of networks would have allowed a more thorough assessment of the relationships among the studied characteristics and also improved our chances of observing self-organized criticality, or of successfully manipulating a network into the critical state by chemical perturbation. This is particularly important in light of the effect observed here of changing the culture media; the branching ratio showed a reliance on the availability of nutrients, especially in the higher-density networks. As stated in Section 4.2, the conventional branching ratio estimate showed cyclic underlying variability with cycle duration equal to the time between media changes, and the MR estimate showed frequent deviations to more subcritical regimes prior to media changes. This suggests the importance of metabolic resources in the networks' ability to maintain consistent levels of activity propagation and complicates the evaluation of trends in the network activity characteristics. Longer or more frequent recordings may also have captured more of the dynamical behavior of the observed networks, and additional GABA perturbations at different points in the network maturity could also reveal variations in the dynamic response to increased inhibition as the networks mature.

This study represents a first step toward understanding the interplay between connectivity and dynamic state that occurs as networks of neurons prepared with different plating densities mature *in vitro*. Studying neurons *in vitro* allows us a level of observation and control not possible *in vivo*, and observing what features arise from their self-organization can give insight into whether information propagation is a component driving the formation of networks in the brain.

## Data availability statement

The raw data supporting the conclusions of this article will be made available by the authors, without undue reservation.

## Author contributions

KH conceptualized the study, contributed to experimental planning, conducted the dynamics analysis, contributed to the connectivity analysis, interpreted the analytical results, and drafted the manuscript. OH planned the experimental details of the study, conducted the connectivity and graph theoretical analysis, provided support in interpreting the analytical results, and aided in drafting the manuscript. VF conducted the experiments and collected data. AS, IS, and SN contributed to and supervised the study design, provided experienced insight in interpreting the analytical results, and revised the manuscript critically for important intellectual content. All authors aided in revising the drafted manuscript and approved the final version.

## Funding

This work was partially funded by the SOCRATES project (Research Council of Norway, IKTPLUSS grant agreement 270961), the DeepCA project (Research Council of Norway, Young Research Talent grant agreement 286558), the Joint Research Committee between St Olav's Hospital and the Faculty of Medicine, NTNU (FFU), the Liaison Committee for Education, Research, and Innovation in Central Norway (Samarbeidsorganet HMN-NTNU), and Enabling Technologies NTNU.

## Conflict of interest

The authors declare that the research was conducted in the absence of any commercial or financial relationships that could be construed as a potential conflict of interest.

## Publisher's note

All claims expressed in this article are solely those of the authors and do not necessarily represent those of their affiliated organizations, or those of the publisher, the editors and the reviewers. Any product that may be evaluated in this article, or claim that may be made by its manufacturer, is not guaranteed or endorsed by the publisher.
